# Effects of acute static, ballistic, and PNF stretching exercise on the muscle and tendon tissue properties

**DOI:** 10.1111/sms.12725

**Published:** 2016-07-01

**Authors:** A. Konrad, S. Stafilidis, M. Tilp

**Affiliations:** ^1^ Sport Science Graz University Graz Austria; ^2^ Faculty of Physical Education and Sport Sciences Aristotle University of Thessaloniki Thessaloniki Greece

**Keywords:** Stiffness, ultrasound, stretching, passive resistive torque, MVC, range of motion

## Abstract

The purpose of this study was to investigate the influence of a single static, ballistic, or proprioceptive neuromuscular facilitation (PNF) stretching exercise on the various muscle‐tendon parameters of the lower leg and to detect possible differences in the effects between the methods. Volunteers (*n* = 122) were randomly divided into static, ballistic, and PNF stretching groups and a control group. Before and after the 4 × 30 s stretching intervention, we determined the maximum dorsiflexion range of motion (RoM) with the corresponding fascicle length and pennation angle of the gastrocnemius medialis. Passive resistive torque (PRT) and maximum voluntary contraction (MVC) were measured with a dynamometer. Observation of muscle‐tendon junction (MTJ) displacement with ultrasound allowed us to determine the length changes in the tendon and muscle, respectively, and hence to calculate stiffness. Although RoM increased (static: +4.3%, ballistic: +4.5%, PNF: +3.5%), PRT (static: −11.4%, ballistic: −11.5%, PNF: −13,7%), muscle stiffness (static: −13.1%, ballistic: −20.3%, PNF: −20.2%), and muscle‐tendon stiffness (static: −11.3%, ballistic: −10.5%, PNF: −13.7%) decreased significantly in all the stretching groups. Only in the PNF stretching group, the pennation angle in the stretched position (−4.2%) and plantar flexor MVC (−4.6%) decreased significantly. Multivariate analysis showed no clinically relevant difference between the stretching groups. The increase in RoM and the decrease in PRT and muscle‐tendon stiffness could be explained by more compliant muscle tissue following a single static, ballistic, or PNF stretching exercise.

Stretching is generally divided into static, ballistic, and proprioceptive neuromuscular facilitation (PNF) stretching (Magnusson et al., 1996). All the methods are used to acutely (a single stretching exercise for several seconds/minutes) increase the range of motion (RoM) (Magnusson, 1998; Herda et al., 2013; Kay et al., 2015). Besides RoM, several other functional [maximal isometric torque, muscle‐tendon stiffness, passive resistive torque (PRT)] or structural parameters (muscle stiffness, tendon stiffness, fascicle length, pennation angle), which might be able to explain the functional changes, may be altered by the use of different stretching methods. In addition to the increased RoM, there is evidence that muscle‐tendon stiffness (Morse et al., 2008; Kato et al., 2010; Nakamura et al., 2011; Kay et al., 2015) and PRT (Kay & Blazevich, 2009; Nakamura et al., 2013) decrease following acute static stretching. Regarding ballistic stretching, Herda et al. (2013) reported decreased PRT following a single stretch. Kay et al. (2015) found decreased muscle‐tendon stiffness following a single PNF stretching exercise. With regard to the studies in the last decade (Morse et al., 2008; Kay & Blazevich 2009; Kato et al., 2010; Nakamura et al., 2011; Herda et al., 2013; Nakamura et al., 2013; Kay et al., 2015), there seems to be evidence that passive forces decrease following an acute stretching exercise, regardless of the stretching method. Despite these results, studies from the 1990s also showed no changes in muscle‐tendon stiffness at a fixed angle following a single static (Halbertsma et al., 1996; Magnusson et al., 1996; Magnusson, 1998), ballistic (Magnusson, 1998), or PNF (Magnusson et al., 1996) stretch. Besides investigations on passive conditions, there are several findings of active measurements on the muscle‐tendon unit (MTU). Concerning maximal isometric contraction movements following single stretching exercises, the literature again provides controversial results. While several studies showed no detrimental effect on maximum performance (static: Kubo et al., 2001; Stafilidis & Tilp, 2015; Kay et al., 2015; ballistic: Herda et al., 2008; PNF: Kay et al., 2015), others showed decreased performance (static: Herda et al., 2008; Marek et al., 2005; ballistic: Herda et al., 2013; PNF: Marek et al., 2005) following a single stretching exercise. These controversial results could possibly be explained by the differences in overall stretch duration, as reported in the review by Kay and Blazevich (2012). Several studies in the last decade investigated possible alterations of the muscle‐tendon structure that might explain changes of the muscle‐tendon function [e.g., RoM, PRT, maximum voluntary contraction (MVC)] following acute stretching exercises. There have been conflicting reports about the effects of acute static stretching on the muscular and tendinous components of the MTU. While Kay and Blazevich (2009) and Kay et al. (2015) reported a decrease in stiffness of the muscle component, Kubo et al. (2001) and Kato et al. (2010) reported decreased tendon stiffness. Moreover, Nakamura et al. (2011) reported decreased muscle stiffness and increased tendon stiffness. Furthermore, they found no changes in fascicle length following a static stretch. Regarding ballistic stretching, Samukawa et al. (2011) found no changes in pennation angle and fascicle length following a single stretch. However, they noted a distal displacement of the muscle‐tendon junction (MTJ), and therefore concluded that changes in the tendon tissue had occurred. Concerning a single PNF stretching exercise, Kay et al. (2015) found decreased muscle as well as tendon stiffness. To the best of our knowledge, only Kay et al. (2015) have compared the acute effects of different stretching techniques (static and PNF) on structural parameters with an identical method. The authors found decreased tendon stiffness in the PNF stretching group and decreased muscle stiffness in both the static and PNF stretching groups. However, the changes in muscle stiffness did not differ between the groups.

Nevertheless, no studies have determined the functional and structural parameter changes in all three common stretching methods, namely static, ballistic, and PNF stretching, on the MTU with the same setup. Thus, this study is the first that has examined and compared the effects of all three main stretching methods on the functional (RoM, PRT, MVC, muscle‐tendon stiffness) and structural (muscle and tendon stiffness in passive conditions, tendon stiffness in active conditions) parameters of the lower leg muscles.

Therefore, the main objective of this study was to analyze the effects of a single stretching exercise intervention with the three main stretching methods on the functional and structural parameters of the plantar flexor MTU. Furthermore, a secondary objective was to determine if there was any difference between the effects of the different stretching methods. Due to the findings in the literature, we hypothesized a gain in RoM and adaptations in the MTU (e.g., more compliant tendon and/or muscle tissue) with all three stretching techniques. Moreover, due to the different forces on the MTU of the examined stretching methods, we expected different functional and structural adaptations following the three stretching interventions.

## Materials and methods

### Experimental design

A total of 122 subjects participated in the study. The intervention groups (police cadets) were randomly assigned to a static stretching group (*N* = 25), a ballistic stretching group (*N* = 24), and a PNF stretching group (*N* = 49) by picking cards in a blind manner. The control group (*N* = 24; students of sport science) was tested separately. The different makeup of the intervention groups and the control group was due to the fact that the intervention groups also took part in a longitudinal study (Konrad & Tilp, 2014a, b; Konrad et al., 2015). For the acute effects of stretching, a control group was tested later on a separate occasion to complete the overall experiment. Before and after the stretching intervention, the RoM, PRT, MVC, and several parameters of the MTU (fascicle length, pennation angle, muscle stiffness, passive and active tendon stiffness) of the gastrocnemius medialis (GM) were determined.

### Subjects

Seventy‐nine healthy male (mean ± SD; 23.3 ± 2.5 years, 180.2 ± 5.7 cm, 77.2 ± 7.4 kg) and 43 healthy female (mean ± SD; 23.4 ± 3.7 years, 169.7 ± 4.7 cm, 62.0 ± 5.6 kg) subjects participated in this study. The baseline characteristics of all the stretching groups and the control group are shown in Table 1. Each subject was informed about the testing procedure, but not about our hypotheses, and they each gave written consent to participate in the study. In addition to a written introduction, subjects were personally informed about the procedure. Competitive athletes and participants with a history of lower leg injuries were excluded. The Ethical Committee of the University of Graz approved the study.

**Table 1:** Baseline characteristics of the static, ballistic, and PNF stretching groups (without dropout subjects)

	Static	Ballistic	PNF	*P*
Range of motion (°)	30.9 ± 5.2	32.9 ± 5.9	31.4 ± 7.1	0.59
Fascicle length at rest (cm)	6.2 ± 0.8	6.4 ± 0.7	6.2 ± 0.8	0.59
Fascicle length in stretching position (cm)	7.3 ± 0.8	7.4 ± 0.9	7.2 ± 0.8	0.61
Pennation angle at rest (°)	18.9 ± 2.3	17.4 ± 2.2	18.3 ± 1.8	0.06
Pennation angle in stretching position (°)	15.5 ± 1.8	15.4 ± 1.9	16.3 ± 1.7	0.11
Passive resistive torque (Nm)	23.5 ± 7.7	25.9 ± 8.6	23.9 ± 7.6	0.57
Passive tendon stiffness (N/mm)	13.2 ± 4.2	12.7 ± 3.5	12.9 ± 4.4	0.93
Muscle stiffness (N/mm)	7.5 ± 2.5	9.1 ± 3.7	6.9 ± 2.3	0.03^a^
Muscle‐tendon stiffness (Nm/°)	0.77 ± 0.17	0.82 ± 0.22	0.78 ± 0.19	0.72
MVC torque (Nm)	96.7 ± 35.9	86.5 ± 39.9	99.9 ± 41.8	0.44
Active tendon stiffness (N/mm)	24.3 ± 8.3	18.9 ± 3.9	21.7 ± 9.4	0.10

a Significant difference in the baselines between the ballistic and PNF stretching groups, mean ± SD.

### Measures

To ensure a high scientific standard, all measurements were undertaken by the same investigator. The temperature in the laboratory was kept constant at around 20.5 °C. Measurements were performed without any warm‐up and in the following order: (a) RoM (10‐min break); (b) PRT (1‐min break); (c) MVC (1‐min break); (d) stretching regime (1‐min break); (e) PRT (1‐min break); (f) MVC (2‐min break); (g) RoM.

#### Range of motion measurement

Dorsiflexion RoM was measured with an electronic goniometer (Biovision, Wehrheim, Germany) fixed to the ankle joint with Leukotape^®^ (BSN medical S.A.S., Vibraye, France). Participants were first instructed to stay upright in a neutral position, with the ankle joint angle at 90°. They were then asked to step back with one leg and bring the ankle joint to maximum dorsiflexion, keeping their heel on the ground. The knee of the testing leg had to remain fully extended, and the knee of the opposite leg flexed. Both feet were kept in a parallel position, and hands could be placed on a wall to ensure balance. Special attention was given to the appropriate position of the stretched leg during the measurement to avoid any pronation of the foot. If some pronation was observed, the measurement was repeated. The difference between the maximum dorsiflexion and the position in rest (neutral position) was defined as the dorsiflexion RoM (Konrad & Tilp, 2014a, b; Konrad et al., 2015).

#### Passive resistive torque measurement

To investigate PRT, an isokinetic dynamometer (CON‐TREX MJ, CMV AG, Duebendorf, Switzerland) was used, and the standard setup for ankle joint movement of the dynamometer was adjusted. Subjects lay prone with their knee fully extended on a bench, and were secured with a strap on the upper body to exclude any evasive movement. The foot was fixed barefooted with a strap to the foot plate of the dynamometer, and the ankle joint center was carefully aligned with the axis of the dynamometer to avoid any heel displacement. The dynamometer moved the ankle joint from a 10° plantar flexion to a dorsiflexion position, which corresponded to 95% of the individual maximum dorsiflexion RoM previously determined in the RoM measurement. The ankle joint was moved passively for three cycles. During pilot measurements, we recognized a conditioning effect during the first two passive movements, similar to the active conditioning reported by Maganaris (2003). Therefore, measurements were taken during the third cycle to avoid any conditioning effect. Similar to the studies by Kubo et al. (2002) and Mahieu et al. (2009), the velocity of the dynamometer was set at 5°/s to exclude any reflexive muscle activity. Participants were asked to relax during the measurements.

#### Maximum voluntary contraction measurement

MVC measurements were performed with the dynamometer at a neutral ankle position (90°). Participants were instructed to perform three isometric MVCs of the plantar flexors with maximum explosive effort for 5 s, with rest periods of at least 1 min between the measurements to avoid any fatigue. The attempt with the highest MVC value was taken for further analysis.

#### Electromyography (EMG)

Muscular activity was monitored by EMG (myon 320, myon AG, Zurich, Switzerland) during PRT and MVC measurements. Surface electrodes (Blue Sensor N, Ambu A/S, Ballerup, Denmark) were placed on the muscle bellies of the GM and the tibialis anterior. In the PRT measurement, the EMG (normalized to plantar flexor MVC) was monitored post‐hoc to ensure that the subject was relaxed, i.e., did not show EMG activity exceeding 5% of MVC (Gajdosik et al., 2005; Kato et al., 2010). The sample rate was 2000 Hz. The EMG signals were high‐pass filtered (10 Hz, Butterworth) and root‐mean‐square (RMS, 50 ms window) values were calculated.

#### Measurement of elongation of the muscle‐tendon structures

A real‐time ultrasound apparatus (mylab 60, Esaote S.p.A., Genova, Italy) with a 10‐cm B‐mode linear‐array probe (10 MHz; LA 923, Esaote S.p.A., Genova, Italy) was used to obtain longitudinal ultrasound images of the GM. The ultrasound images were recorded at 25 Hz.

**Figure 1:**
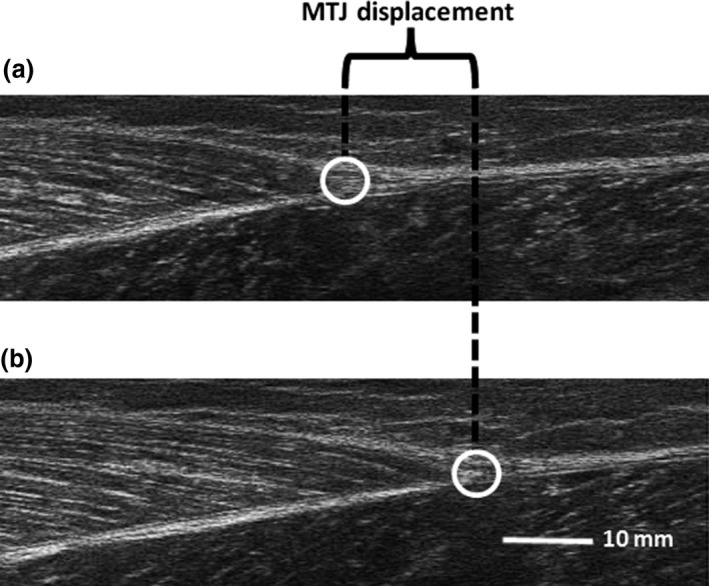
Images showing the displacement of the MTJ during a passive movement from neutral position (a) of the ankle joint to maximum dorsiflexion (b).

During the PRT and MVC measurements, the ultrasound probe was placed on the distal end of the GM (Fig. 1), where the muscle is connected to the Achilles tendon, i.e., the MTJ (Kato et al., 2010). The ultrasound probe was secured with a standard orthopedic stocking to prevent displacement of the probe. To determine the muscle displacement during PRT measurement, the echoes of the MTJ in the ultrasound videos were manually tracked (Kato et al., 2010). During the MVC measurements, the ultrasound probe sometimes lost skin contact above the MTJ due to the deformation of the muscle which led to minor quality of this area in the videos. Thus, the muscle displacement was determined by manually tracking the echoes of a fascicle insertion at the deep aponeurosis near the MTJ (Kubo et al., 2002).

During RoM measurement, the length of the GM fascicle and its pennation angle with the deep aponeurosis were determined from the ultrasound videos. The ultrasound probe was placed at 50% of the GM muscle length (Morse et al., 2008). The fascicle length and the pennation angle were measured at a neutral position of the ankle joint (90°) and at maximum dorsiflexion.

The ultrasound images were recorded at 25 Hz, with an image depth resolution of 74 mm. During PRT and MVC measurements, the videos were synchronized with the rest of the data via the signals of a function generator (Voltkraft^®^, Hirschau, Germany). The videos were cut and digitized in VirtualDub open‐source software (version 1.6.19, www.virtualdub.org) and were analyzed in ImageJ open‐source software (version 1.44p, National Institutes of Health, USA).

Each video was measured by two investigators, and the mean values of both measurements were used for further analysis of the muscle‐tendon structure. Except for the principal investigator, the investigators were not informed of the hypotheses of the study or the group allocation and subjects' names. During the analysis of the PRT measurement, every fifth frame (and for MVC measurement, every second frame) was measured by the investigators, corresponding to a time resolution of 0.2 and 0.08 s, respectively. Similar to the approach used by other authors (Morse et al., 2008; Kato et al., 2010), the cadaveric regression model of Grieve et al. (1978) was used to obtain the length changes of the MTU of the GM during passive movements. The difference between the MTU length change and the displacement of the muscle was defined as the tendon displacement.

#### Calculation of muscle/tendon force, passive muscle/tendon stiffness, active tendon stiffness, and muscle‐tendon stiffness

The muscle force of the GM was estimated by multiplying the measured torque by the relative contribution of the physiological cross‐sectional area (18%) of the GM within the plantar flexor muscles (Kubo et al., 2002; Mahieu et al., 2009), and dividing by the moment arm (MA) of the triceps surae muscle, which was measured by tape measure as the distance between the malleolus lateralis and the Achilles tendon at rest (neutral position; Konrad & Tilp, 2014a, b; Konrad et al., 2015). The mean value of the MA was 4.5 cm and the range was 3.0–6.0 cm.

Active tendon stiffness was calculated by linear regression between the active force and the related tendon length changes during the MVC measurements over the whole range of force (0–100% MVC) at neutral ankle position (90°; Konrad & Tilp, 2014a, b; Konrad et al., 2015). Passive tendon stiffness, muscle stiffness, and muscle‐tendon stiffness were calculated as the change in the passive force produced from the neutral ankle position (90°) to maximum dorsiflexion (before stretching) divided by the change of the related tendon length, muscle length, and joint angle, respectively. The quality of the linear regressions was assessed with the Pearson's correlation coefficients (Konrad & Tilp, 2014a, b; Konrad et al., 2015).

### Stretching interventions

The stretching for all the techniques was undertaken with the dynamometer. Starting at neutral ankle position (90°), the dynamometer was moved to the maximum dorsiflexion RoM of the subjects in all stretching techniques. In the static stretching group, the intervention consisted of a single 30‐s static stretch of the lower leg at the maximum dorsiflexion RoM. During the ballistic stretching, a dynamic movement at a frequency of 1 Hz (Mahieu et al., 2007) at the last 5° of the subjects' individual RoM was undertaken by the dynamometer to stretch the MTU passively. Subjects of the PNF stretching group were asked to undertake a “contract‐relax‐antagonist‐contract” PNF stretching intervention (Sharman et al., 2006) for the plantar flexor muscles at the maximum dorsiflexion RoM. This consisted of a 15‐s passive static stretch of the lower leg followed by a submaximal isometric contraction (~80% of the MVC) of the stretched plantar flexor muscles for 6 s. Afterwards, the subjects were instructed to contract the antagonistic dorsi flexor muscles for another 15 s (Mahieu et al., 2009) to induce another stretch for the plantar flexors. The static, ballistic, and PNF procedures were repeated four times during the stretching session, with a rest of 20 s (in the neutral ankle position) in between, resulting in a total stretch period of 120 s for the triceps surae muscle. This protocol was chosen because it has been reported that 4 × 30 s of static stretching can decrease MTU stiffness (Ryan et al., 2008). The control group did not receive any intervention during the stretching session and had a rest of 4 min in the prone position between the pre‐ and post‐measurements.

### Statistical analyses

**Figure 2:**
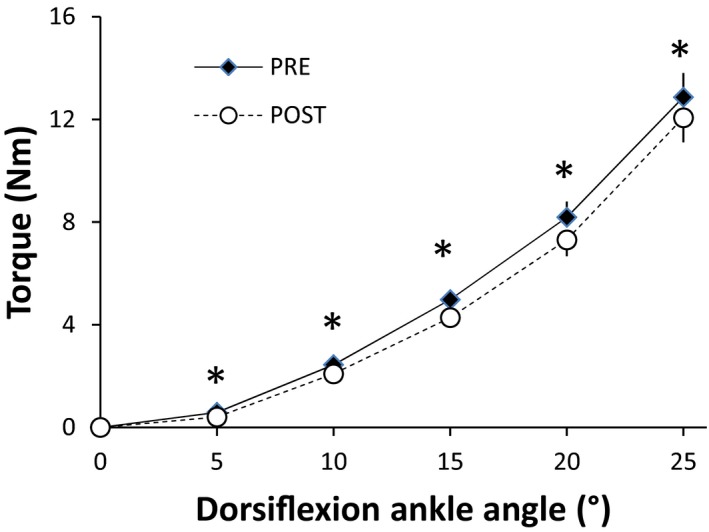
Relationship between PRT and ankle joint angle before and after the static stretching intervention (*N* = 17), mean (standard error of mean). 0° represents neutral ankle position. *Significant difference between pre‐ and post‐session data.

SPSS (version 20.0, SPSS Inc., Chicago, Illinois, USA) was used for all the statistical analyses. To determine the inter‐rater reliability of the muscle‐tendon displacement measurements, intraclass correlation coefficients (ICCs) were used. A Kolmogorov–Smirnov test was used to verify the normal distribution of all the parameters. To prove the homogeneity between the baselines characteristic of the intervention groups, a one‐way ANOVA test was performed. To verify possible gender differences and possible differences in the effects of stretching, unpaired *t*‐test subsequently were performed (see discussion section; Table 4). To check our measurement methods, paired *t*‐tests were performed to test if the mean values of the pre‐ and post‐measurements of the control group were different. To demonstratively test the effect of the static stretching protocol on the torque‐angle curve at 5°, 10°, 15°, 20°, and 25° (see Fig. 2), a repeated‐measures ANOVA test with post‐hoc paired *t*‐tests for the different angles was performed. Subsequently, the outcomes of the three different stretching techniques were compared to assess whether there was any difference in the effects of these techniques. To do this, a MANOVA test and a post‐hoc test with a Bonferroni correction was performed to assess the difference between the three stretching groups (static stretching, ballistic stretching, PNF stretching). In the case of a linear relationship between variables, a MAN(C)OVA test was performed. Considering the suggestion of Olson (1976), the Pillai's Trace *P*‐value was taken for further consideration regarding the MAN(C)OVA test. The linearity of the tendon and muscle force–length relationship and the muscle‐tendon moment–angle relationship for the stiffness calculations (which was previously described in the ‘calculations of the stiffness’) was controlled with the Pearson's correlation coefficients. An alpha level of *P* = 0.05 was defined for the statistical significance of all the tests.

## Results

### Data exclusion and measurement quality

Several subjects had to be excluded from the study (see Table 2) due to the poor quality of the ultrasound videos. In the ultrasound videos with a poor quality, the fascicle insertion points at the deep aponeurosis (MVC measurement) or the MTJ (PRT measurement) were not identifiable with the necessary precision.

**Table 2:** Group and gender allocation with anthropometrics and data exclusion in all groups due to the poor quality of the ultrasound videos. Moreover, the numbers of subjects used in the statistical analysis is illustrated in this table

	Static	Ballistic	PNF	Controls
Number of subjects	25	24	49	24
Male/female	21/4	16/8	31/18	11/13
Age [years] (mean ± SD)	23.3 ± 3.2	22.6 ± 2.8	23.5 ± 2.7	23.8 ± 3.5
Height [cm] (mean ± SD)	177.9 ± 5.5	177.0 ± 8.2	176.6 ± 6.8	174.1 ± 8.9
Weight [kg] (mean ± SD)	74.3 ± 9.3	72.2 ± 10.0	72.1 ± 10.1	68.2 ± 9.6
Dropouts				
RoM	4	3	10	1
Passive	3	4	18	0
Active	3	3	8	1
Subjects further analyzed				
RoM	21	21	39	23
Passive	22	20	31	24
Active	22	21	41	23

To assess the inter‐rater reliability of the measurements, ICC (3,k) was calculated. The mean (range of all videos) ICC values were 0.99 (0.989–0.997), 0.98 (0.976–0.990), 0.96 (0.801–0.999), and 0.96 (0.801–0.999) for pennation angle and fascicle length during RoM measurement, MTJ displacement during PRT measurement, and MTJ displacement during MVC measurement, respectively. Values above 0.90 are classified as high (Vincent & Weir, 2012).

The mean values of the Pearson's correlation coefficients at the linear regression were 0.98, 0.96, 0.90, and 0.96, with ranges of 0.88–0.99, 0.65–0.99, 0.82–0.97, and 0.92–0.98, with all *P* < 0.05, for calculations of passive tendon stiffness, active tendon stiffness, muscle stiffness, and muscle‐tendon stiffness, respectively (Calculations of linearity was previously described in the ‘calculations of the stiffness’).

### Range of motion and the related structural muscle parameters

Following the stretching intervention, all the stretching groups had a significantly increased dorsiflexion RoM (*P* < 0.05, see Table 3a). Furthermore, the pennation angle decreased significantly only in the PNF stretching group in the maximum dorsiflexion position (*P* = 0.01), but not in the neutral position. Fascicle length did not change in either position. No parameter changes were observed in the control group. The MANCOVA test (dependent variables: RoM, fascicle length in stretching position, pennation angle in stretching position. Covariates: fascicle length at rest and pennation angle at rest) showed a significant group effect (*P* = 0.00; *F* = 3.855; df = 150). The post‐hoc *t*‐tests revealed a significant difference in the pennation angle in the stretching position between the static and PNF stretching groups.

**Table 3:** (a) Results of maximum dorsiflexion RoM, as well as changes in fascicle length and pennation angle during RoM measurement. (b) Results of PRT, passive tendon stiffness, muscle stiffness, and muscle‐tendon stiffness during passive measurements. (c) Results of MVC torque and active tendon stiffness during MVC measurements

	Static^b^			Ballistic			PNF			Controls		
	PRE	POST	*P*	PRE	POST	*P*	PRE	POST	*P*	PRE	POST	*P*
(a)	*N* = 21			*N* = 21			*N* = 39			*N* = 23		
Range of motion (°)	30.9 ± 5.2	32.3 ± 6.3	0.03^a^	32.9 ± 5.9	34.4 ± 5.9	0.04^a^	31.4 ± 7.1	32.5 ± 7.2	0.02^a^	34 ± 6.6	34.7 ± 6.9	0.44
Fascicle length at rest (cm)	6.2 ± 0.8	6.1 ± 0.9	0.76	6.4 ± 0.7	6.4 ± 0.8	0.55	6.2 ± 0.8	6.1 ± 0.7	0.25	6.1 ± 0.7	6.1 ± 0.7	0.60
Fascicle length in stretching position (cm)	7.3 ± 0.8	7.4 ± 0.9	0.31	7.4 ± 0.9	7.3 ± 0.8	0.48	7.2 ± 0.8	7.2 ± 0.7	0.48	7.3 ± 0.9	7.3 ± 0.9	0.65
Pennation angle at rest (°)	18.9 ± 2.3	18.4 ± 2.1	0.08	17.4 ± 2.2	17.4 ± 1.8	0.98	18.3 ± 1.8	18.5 ± 1.7	0.67	18.3 ± 1.7	18.2 ± 1.8	0.62
Pennation angle in stretching position (°)	15.5 ± 1.8	15.7 ± 1.7	0.44	15.4 ± 1.9	15.3 ± 1.7	0.84	16.3 ± 1.7	15.6 ± 1.4	0.01^a^	16 ± 1.6	15.7 ± 1.6	0.46
(b)	*N* = 22			*N* = 20			*N* = 31			*N* = 24		
Passive resistive torque (Nm)	23.5 ± 7.7	20.8 ± 7.5	0.00^a^	25.9 ± 8.6	22.9 ± 7.7	0.00^a^	23.9 ± 7.6	20.6 ± 7.9	0.00^a^	20.4 ± 8.3	19.9 ± 8.6	0.13
Passive tendon stiffness (N/mm)	13.2 ± 4.2	12.8 ± 5.3	0.60	12.7 ± 3.5	13.4 ± 4.3	0.27	12.9 ± 4.4	11.6 ± 5.3	0.11	9.1 ± 2.8	8.9 ± 2.3	0.34
Muscle stiffness (N/mm)	7.5 ± 2.5	6.5 ± 2.6	0.04^a^	9.1 ± 3.7	7.3 ± 2.4	0.01^a^	6.9 ± 2.3	5.6 ± 1.9	0.00^a^	6.3 ± 2.2	6.2 ± 2.5	0.67
Muscle‐tendon stiffness (Nm/°)	0.77 ± 0.17	0.69 ± 0.18	0.00^a^	0.82 ± 0.22	0.73 ± 0.20	0.00^a^	0.78 ± 0.19	0.67 ± 0.22	0.00^a^	0.61 ± 0.19	0.6 ± 0.21	0.23
(c)	*N* = 22			*N* = 22			*N* = 41			*N* = 23		
MVC torque (Nm)	96.7 ± 35.9	97 ± 37.8	0.88	86.5 ± 39.9	84.9 ± 36.6	0.44	99.9 ± 41.8	95.4 ± 39.1	0.01^a^	69.7 ± 33.0	70.3 ± 35.4	0.81
Active tendon stiffness (N/mm)	24.3 ± 8.3	21.9 ± 7.2	0.06	18.9 ± 3.9	19.2 ± 4.4	0.80	21.7 ± 9.4	21.6 ± 10.6	0.96	17 ± 5.5	17.2 ± 6.4	0.81

a Significant difference between pre‐ and post‐session data.

b Significant difference between the static and PNF stretching groups at the RoM measurement and its corresponding parameters, mean ± SD.

### Passive resistive torque and related structural muscle‐tendon parameters

**Figure 3:**
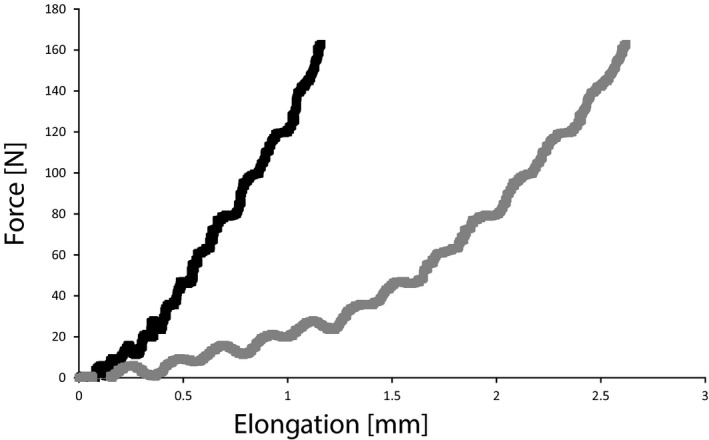
Force elongation curve during a passive movement of the ankle angle. The gray curve shows the force elongation curve of the muscle and the black curve shows the force elongation of the tendon.

There was a significant decrease in PRT at the same maximum ankle joint angle, in muscle‐tendon stiffness and muscle stiffness, in all stretching groups from the pre‐ to the post‐session data. No significant differences were observed in the passive tendon stiffness. No parameter changes could be found in the control group (see Table 3). Demonstratively, we tested the difference between the pre‐ and post‐session data for the static stretching group. Figure 2 shows that there was a significant difference between the pre‐ and post‐session data at all angles (5°, 10°, 15°, 20°, 25° dorsiflexion). Figure 3 shows exemplary force elongation curves of both muscle and tendon elongations during the passive measurement of one subject. The MANOVA test showed no difference in the effects of the stretching groups.

### Maximum voluntary contraction and tendon stiffness

Plantar flexor MVC significantly decreased in the PNF stretching group following the stretching exercise, but did not change in the other groups. Moreover, there was no significant effect on active tendon stiffness in any group (see Table 3c). The MANOVA test showed no difference in the effects of the different stretching groups.

## Discussion

The aims of this study were to determine the possible functional and structural changes caused by different stretching methods and to analyze the possible differences in the effects of the methods. The functional parameters investigated in this study were RoM, PRT, muscle‐tendon stiffness, and MVC. In the following, each parameter is discussed in turn.

### Range of motion

The maximum dorsiflexion RoM increased following each of the different techniques. This is in accordance with our hypothesis and the results of similar studies incorporating a single static (Morse et al., 2008; Kato et al., 2010; Kay et al., 2015), ballistic (Herda et al., 2013), or PNF stretching regime (Kay et al., 2015). However, the amount of dorsiflexion RoM increase in the present study (static: 1.4° ballistic: 1.5° PNF: 1.1°) was less than for other studies [static: 4.6° for Morse et al. (2008) , 9° for Kato et al. (2010), 2.6° for Kay et al. (2015), PNF: 5.3° for Kay et al. (2015)].

In some studies of static stretching, the greater changes in RoM could possibly be explained by the greater stretch durations used. Kato et al. (2010) used a continuous stretch duration of 20 min and Morse et al. (2008) stretched the subjects five times for 1 min, resulting in a total stretching duration of 5 min compared to the total stretch duration of 2 min in the present study. However, our results were in the range of results obtained by Kato et al. (2010) following a static stretching over 5 min (2°), and were similar to the values presented by Kay et al. (2015), who stretched the subjects for 1 min in total. Thus, it seems that the stretch duration is positively related to the gains in RoM in static stretching.

A further reason for the lower yields in RoM in the present study in all the stretching groups could be that the stretches were performed at a constant angle, whereas in the studies of Kato et al. (2010) and Morse et al. (2008), the subjects stretched at a constant torque value. Kay et al. (2015) stretched with constant angle, however, following every bout the adapted RoM was considered for the following stretch. During the first bout of stretching, our subjects stretched until the point of discomfort at their individual maximum dorsiflexion RoM. From the second to the fourth bout, the dynamometer was moved to the same angle as the first bout. However, with regard to the latest results by Herda et al. (2014, see their fig. 1), one could assume that, in these bouts, the maximum achievable dorsiflexion might not be reached with our method. Thus, the subjects of Morse et al. (2008) and Kato et al. (2010) might have received a higher stretch stimulus by the constant torque approach (Cabido et al., 2014). Moreover, the subjects of Kay et al. (2015) possibly received a higher stretch stimulus due to the adapted RoM following every stretching bout. A further reason for the small increase in RoM could lie in the MVCs and the elapsed time following the stretching regime. Moreover, the elapsed time from the stretching regime to the RoM measurement in our study was approximately 11 min. Although an increase in RoM has been reported to last more than 30 min following 5 × 1 min of stretching (Mizuno et al., 2013), this could have affected the magnitude of the RoM changes in our experiments. Nevertheless, the RoM significantly increased in all the stretching groups.

### Passive resistive torque and muscle‐tendon stiffness

It can be shown for the first time that in a single study with the same methods, the muscle‐tendon stiffness and PRT decreased following all the stretching interventions. This finding is similar to those reported by previous studies dealing with static [muscle‐tendon stiffness: (Morse et al., 2008; Nakamura et al., 2011; Kato et al., 2010; Kay et al., 2015); PRT: (Kay & Blazevich, 2013)], ballistic (PRT: Herda et al., 2013), or PNF stretching (muscle‐tendon stiffness: Kay et al., 2015) regimes. However, Magnusson (1998) reported unchanged muscle‐tendon stiffness at fixed angles and significantly increased muscle‐tendon stiffness at the adapted angle with the static and ballistic stretching exercises. Although Magnusson (1998) examined the hamstring muscles, the other studies (Morse et al., 2008; Kay & Blazevich, 2009; Kay et al., 2015; Kato et al., 2010) examined the effects of acute static stretching on the lower leg muscles. Therefore, one could speculate that the hamstring muscles may change differently following an acute stretching exercise compared to the lower leg muscles, e.g., because of the different muscle architectures. The observed changes in PRT and muscle‐tendon stiffness strengthen the hypothesis that increased stretch tolerance (Magnusson, 1998; Kay et al., 2015) is not exclusively responsible for the increase in RoM.

### Maximum voluntary contraction

Concerning the functional parameter MVC, only in the PNF stretching group was there a detrimental effect following the stretching regime, while the maximum isometric torque of the static and ballistic stretching groups did not change. However, contradictory results can be found in the literature regarding this topic. Although several studies showed no detrimental effect on maximum performance (static: Kubo et al., 2001; Stafilidis & Tilp, 2015; Kay et al., 2015; ballistic: Herda et al., 2008; PNF: Kay et al., 2015), others reported a decreased performance (static: Herda et al., 2008; Marek et al., 2005; ballistic: Herda et al., 2013; PNF: Marek et al., 2005) following a single stretching exercise. The loss in maximal isometric torque in the PNF stretching group in the present study and in the studies of Marek et al. (2005) could possibly be explained by the contraction and the resulting fatigue of the target muscles during the stretching regimes. However, Kay et al. (2015) reported unchanged maximum torque values following a single PNF stretching exercise, as well as a single static stretching exercise. While Kay et al. (2015) used the contract relax (CR) technique, we used the CRAC (contract‐relax‐antagonist‐contract) method in the present study. In the CR method, the target muscle is placed into a stretch position followed by an isometric contraction of the target muscle in the stretching position. The CRAC method includes a further isometric contraction of the antagonist muscle, which might have influenced the MVC.

In addition to the functional parameters, several structural parameters (muscle stiffness, tendon stiffness, fascicle length, pennation angle), which might help to explain the increased RoM, the decreased PRT, and muscle‐tendon stiffness, were investigated in this study. In all the stretching groups, muscle stiffness decreased following the stretching regime; however, the passive and active tendon stiffness remained unchanged.

### Muscle stiffness and tendon stiffness

Decreased muscle stiffness and unchanged tendon stiffness following a static stretching exercise was also reported by Kay and Blazevich (2009) and Kay et al. (2015). Others, however, reported decreased tendon stiffness (Kubo et al., 2001; Kato et al., 2010) and unchanged muscle stiffness (Kato et al., 2010) following a single static stretch. One possible explanation for these contradictory results observed after an acute static stretching exercise could again be the duration of the stretching exercises. Kubo et al. (2001) and Kato et al. (2010) stretched their subjects once for 10 min and 20 min, respectively, and reported decreased tendon stiffness. In contrast, Kay and Blazevich (2009), Kay et al. (2015), and the present study performed repeated static stretches for 2 × 60, 4 × 15, and 4 × 30 s, respectively, and found adaptation in the muscle tissue properties only. Therefore, a conclusion could be that static stretching durations from 60 to 120 s affect the muscle tissue, whereas continuous static stretching for more than 10 min also affects the tendon tissue properties. As the relaxed muscle tissue is more compliant than tendon tissue (Morse et al., 2008; Kato et al., 2010; Kay et al., 2015), one could assume that tendon tissue changes its properties more slowly and therefore later than muscle tissue. Further research on this topic is necessary in order to investigate the possible different changes in muscle‐tendon properties due to different durations of acute static stretching exercise.

The results of the PNF stretching group, where decreased muscle stiffness, but not tendon stiffness, was found, are different to the recent study of Kay et al. (2015). In their study, both muscle and tendon stiffness were found to decrease following PNF‐like stretching. Again, one possible explanation could be the different PNF stretching methods (CR vs CRAC) used. Hence, in the present study, but not in the study of Kay et al. (2015), the antagonist muscle was also contracted. Another reason for the contradictory results could be the stretching duration and the contraction time. While the subjects of Kay et al. (2015) stretched four times for 10 s followed by a 5‐s contraction, our subjects stretched four times for 15 s, contracted isometrically for 6 s, and stretched again for 15 s with a simultaneous antagonistic contraction. Both studies repeated the stretching bouts four times. This resulted in total stretching (contraction) times of 40 (20) and 120 (24) s for Kay et al. (2015) and this study, respectively. It is also likely that a further reason for the different results could be the different stretching intensities used. Kay et al. (2015) stretched at an adapted constant angle level following every bout, while we stretched at a constant angle level. Therefore, one could speculate that due to the higher stimulus of the adapted constant angle stretching, the tendon tissue properties were also affected. A further reason for the different results between Kay et al. (2015) and our study could be in the different contraction intensities used. Although the subjects of Kay et al. (2015) performed maximum isometric contractions, our subjects performed submaximal contractions (~80% of MVC torque). Therefore, it is likely that the subjects of Kay et al. (2015) produced a higher load on the tendon due to the greater contraction intensity. Furthermore, the different calculations of the tendon stiffness could be a possible reason for different results. Although Kay et al. (2015) calculated the tendon stiffness at a range of MVC between 50% and 90%, we calculated tendon stiffness over the whole range of force. Moreover, the MVC of Kay et al. (2015) was performed as a ramped contraction, while our subjects were encouraged to fully contract for 5 s.

### Pennation angle and fascicle length

Similar to our results in the ballistic stretching (and also static stretching) group, Samukawa et al. (2011) also found no changes in pennation angle and fascicle length following a single ballistic stretch. Moreover, Nakamura et al. (2011) reported no changes in pennation angle and fascicle length following a single static stretch for 5 × 1 min. For the PNF stretching group in our study, the fascicle length did not change, although the pennation angle at the stretching position significantly decreased from 16.3° to 15.6° following the stretching regime. One could speculate that this slight difference is possibly due to the more compliant muscle tissue following stretching. However, decreased muscle stiffness was also observed in the static and ballistic stretching group without a significant decrease in the pennation angle. A further speculation to explain the decreased pennation angle in the PNF stretching group without changes in the other stretching groups could be that the antagonist contraction during PNF stretching may have stretched/compressed the plantarflexor muscles more than during the other interventions.

### Comparison of the three stretching techniques

Regarding our first hypothesis, as expected, the RoM increased in all the stretching groups. This increase in RoM can be explained by the more compliant muscle tissue in all the stretching groups. Corresponding to the decrease in muscle stiffness, overall muscle‐tendon stiffness and PRT also decreased in all the stretching groups. In our second hypothesis, we expected different effects in the different stretching groups due to the different forces acting on the tissue of the MTU. Despite a significant difference in fascicle pennation angle between static and PNF stretching, there was little evidence that the different methods led to different effects. This is underlined by the range of mean changes of the pennation angles in stretched positions of −0.7° and +0.2° for the static and PNF groups, respectively, which seems to be marginal.

RoM changes were similar in all the stretching groups. This was surprising because of the previous findings in the literature that showed that a single PNF stretching exercise is more effective than a single static stretch (Sharman et al., 2006) with regard to possible increases in RoM. The most probable explanation appears to be that the constant angle stretch in the dynamometer in all stretching methods and the use of same angle in all subsequent stretches may limit loading of the MTU across the groups. We hypothesize that stretching with constant torque would have led to significant differences between the three stretching methods concerning the RoM.

No differences between the stretching groups regarding the passive/active measurements and the related parameters were observed. To the best of our knowledge, the present study is the first that has compared the effects of the most common stretching techniques on the muscle and tendon tissue properties with the same method. Although we would have expected different changes in the MTU due to the different stretching interventions, no differences with a clinical relevance were observed.

### Limitations

There were some limitations to this study. Firstly, the persons taking measurements were not all blind to the intervention. Therefore, a bias in the results cannot be completely discounted, although the inter‐rater reliability was excellent (mean ICC: 0.96–0.99). Secondly, the method of measuring the MA of the ankle joint *in vivo* was quite simple. However, the values obtained in this study were very similar to others using magnetic resonance imaging data (Rugg et al., 1990) or ultrasound (Lee & Piazza, 2009). The measurement of the MA at rest probably underestimates the MA during MVC by 22–44% (Maganaris, 2004), and also probably in maximum dorsiflexion position, and therefore overestimates tendon force and its related parameters. However, this systematic overestimation would affect all the measurement outcomes, and therefore would not affect the main results of the study. Thirdly, the subjects of the control group showed heterogeneity in several parameters compared to the intervention groups. This could possibly be explained by the higher number of female subjects in the control group (>50%) than the intervention groups (~30%). Furthermore, the subjects of the intervention groups were police cadets, while the control group consisted of students of sports science. This different makeup was due to the fact that the intervention groups also took part in a longitudinal study (Konrad & Tilp, 2014a, b; Konrad et al., 2015). For the acute effects of stretching, a control group was tested later on a separate occasion to complete the overall experiment. However, the main reason for the control group was to check the reliability of our measurements. As the results showed no significant change of all the measured parameters in the control group, reliability was ensured.

Fourthly, we did not take into account gender distribution in our randomization process. Although it is well known that structural and functional parameters are different between the sexes (see e.g., Morse, 2011: higher muscle stiffness in males; see also Table 4 of current study), we expected no differences in the effects due to stretching exercises between the sexes. This assumption was confirmed by a post‐hoc comparison of the effects of stretching between males and females. Except of the MVC torque changes in the static stretching group, no significant difference in the effects of stretching between sexes could be observed. The difference observed in MVC changes in the static stretching group could be explained by the small proportion of females (3 female:19 males) which might have affected the result.

**Table 4:** Comparison of the baseline values between male and female subjects

	Male	Female	*P*
Range of motion (°)	31.6 ± 7.1	32.2 ± 4.9	0.18
Fascicle length at rest (cm)	6.2 ± 0.8	6.1 ± 0.7	0.42
Fascicle length in stretching position (cm)	7.4 ± 0.9	7.1 ± 0.8	0.11
Pennation angle at rest (°)	18.8 ± 1.9	17.3 ± 1.8	0.00^a^
Pennation angle in stretching position (°)	16.2 ± 1.6	15.4 ± 1.8	0.02^a^
Passive resistive torque (Nm)	24.9 ± 8.4	20.5 ± 6.9	0.08^a^
Passive tendon stiffness (N/mm)	12.7 ± 4.2	10.7 ± 3.8	0.02^a^
Muscle stiffness (N/mm)	7.4 ± 2.7	7.2 ± 3.0	0.74
Muscle‐tendon stiffness (Nm/°)	0.81 ± 0.20	0.63 ± 0.17	0.00^a^
MVC torque (Nm)	107 ± 36.1	58.2 ± 23.9	0.00^a^
Active tendon stiffness (N/mm)	23.3 ± 8.2	15.5 ± 3.6	0.00^a^

a Significant difference between the sexes.

Fifthly, the range in which passive tendon and muscle stiffness was calculated was between anatomical zero (90°) to 95% of the RoM in the passive trial. Moreover, during the active trial, the measurement position was also 90°. However, there is evidence that the “real” anatomical zero (where no torque is produced) is not 90° but is rather from 100° to 110° (=10° to 20° of plantarflexion; Riener & Edrich, 1999, their fig. 2). It could be the case that at our measurement position of 90°, the subjects' MTUs were already stretched and, therefore, the stiffness values were overestimated. However, as this systematic overestimation was present both before and after the trials, we expect that it did not affect the principal outcome of this study.

Sixthly, during the PRT and MVC measurement, we only monitored the proximal end of the Achilles tendon. However, the displacement of the distal end of the Achilles tendon, the calcaneal insertion, contributes 54–71% of the MTJ displacement (Seynnes et al., 2015). As our ultrasound probe is not able to detect both ends of the Achilles tendon and the distal end of the muscle belly of the GM, we only could monitor the proximal end of the Achilles tendon and the distal end of the muscle belly. For future studies, it would be useful to add a further ultrasound probe at the calcaneal insertion of the Achilles tendon (Seynnes et al., 2015). However, as we measured the displacement of the MTJ at the proximal end of the Achilles tendon before and after the stretching exercise, we expect that this did not affect the principal outcome of this study.

## Perspectives

This study has shown that a single static, ballistic, or PNF stretching exercise increases dorsiflexion RoM and decreases muscle stiffness. No clinically relevant differences in the effects of stretching between the stretching groups could be detected. Therefore, one could conclude that a single stretching exercise (independent of the method) for four times for 30 s is an appropriate tool to increase the RoM and to decrease muscle stiffness. However, further studies including neurological parameters, which might give additional explanations for possible differences between the stretching exercises, should be undertaken.

## Conflicts of interest

No conflicts of interest are declared by the authors.
